# Epithelial–Mesenchymal Transition Induced by a Metal Mixture in Liver Cells With Antioxidant Barrier Decreased

**DOI:** 10.1155/omcl/6983256

**Published:** 2024-12-18

**Authors:** M. Valverde, P. Rosales-Cruz, E. Torrejon-Gonzalez, A. Ponce-Ortiz, M. A. Rodriguez-Sastre, E. Rojas

**Affiliations:** Instituto de Investigaciones Biomédicas, Universidad Nacional Autonoma de Mexico, Mexico, Mexico

**Keywords:** catalase and GSH abatement, epithelial–mesenchymal transition, metals mixture, wound healing

## Abstract

Occupational exposure to arsenic (As), cadmium (Cd), and lead (Pb) affects many sectors, necessitating research to understand their transformation mechanisms. In this study, we characterized the process of epithelial–mesenchymal transition (EMT) in a rat hepatic epithelial cell line with decreased expression of catalase and glutamate cysteine ligase catalytic (GCLC) subunit that was exposed to a mixture of As, Cd, and Pb at equimolar occupational exposure concentrations. We evaluated the expression of genes and proteins involved in EMT. Our findings revealed that cells with a decreased antioxidant barrier showed a decreased expression and abundance of epithelial genes when exposed to a mixture of metals. Additionally, we observed alterations in the expression of transcription factors (TFs) associated with EMT and an increase in the expression and abundance of mesenchymal genes. Specifically, we found that E-cadherin expression decreased by ~50% at both the gene and protein levels. In contrast, the expression of *vimentin*, *α-smooth muscle actin*, and *N-cadherin* genes increased by ~70%, whereas their corresponding protein levels increased by nearly 100%. Furthermore, the TFs zinc finger e-box binding homeobox 1 and snail family transcriptional repressor 1 showed a 30% increase in gene expression and an ~80% increase in protein expression. These changes enable the cells to acquire migratory capabilities. Our results confirmed that exposure to this mixture of As, Cd, and Pb can induce EMT in cells with a decreased antioxidant barrier.

## 1. Introduction

With the development of industry and urbanization, heavy metal contamination has become an important issue, affecting both organisms and ecosystems owing to their high toxicity. Humans are frequently exposed to mixtures of metals, primarily arsenic (As), cadmium (Cd), and lead (Pb). Epidemiological studies have indicated that populations exposed to metal mixtures exhibit higher cancer-associated mortality rates [1]. Furthermore, the Halifax Project states “…that low-dose exposures to mixtures of chemicals that are not individually carcinogenic may be capable of generating or enabling carcinogenesis [2].” This observation raises concerns that chemical mixtures may act as carcinogens, highlighting the inadequacy of chemical safety studies that do not evaluate these combinations.

Different mechanisms of carcinogenesis have been established for these metals, including an increase in reactive oxygen species (ROS), depletion of the antioxidant system, DNA damage, and various epigenetic processes such as methylation and miRNA induction [3–9]. Recently, the epithelial–mesenchymal transition (EMT) has been increasingly associated with carcinogenesis. EMT enables cells to acquire motility and invasive capabilities comparable to those of transformed cells, challenging the paradigm in which fully differentiated cells remain in a permanent state of differentiation. During EMT, epithelial cells undergo transcriptional reprograming [10–12], principally characterized by the downregulation of genes associated with the epithelial phenotype and upregulation of mesenchymal-related genes, leading to the transdifferentiation of epithelial cells into motile mesenchymal cells.

EMT is a multistage process involving gradual remodeling of cellular architecture and functional capacities within the epithelium [13]. EMT is a conserved program in both developmental and pathological contexts [14]. Key events in this process include the dissolution of cell–cell junctions, loss of apicobasal polarity, acquisition of frontal-distal polarity, reorganization of the cytoskeleton, alterations in cell shape, increased cellular protrusions, enhanced mobility, and often, the ability to degrade the extracellular matrix.

During EMT, genes associated with the epithelial phenotype, such as *Cadherin 1* and *β-catenin* are downregulated, while those related to the mesenchymal phenotype, including *SNAI1*, *ZEB*, vimentin (*VIM*), and *Cadherin 2*, exhibit increased expression. Furthermore, cells that undergo EMT acquire resistance to senescence and apoptosis [11].

Recent studies have shown that various As and Cd compounds are involved in the EMT process, with some promoting EMT and others inhibiting it [15]. However, data on the effects of exposure to mixtures of these compounds at low doses are limited.

To test this hypothesis, in this study, we aimed to understand the role and potential carcinogenic mechanisms of low-concentration metal exposure. We investigated whether the EMT process is crucial in metal-induced transformation in hepatic epithelial cells deficient in glutathione and catalase.

## 2. Material and Methods

### 2.1. Biological Material

The Clone 9 cell line (ATCC CRL-1439), derived from 4-week-old Sprague–Dawley rat liver epithelial cells, was used in this study. A total of 2 × 10^5^ cells were seeded in p100 dishes containing 10 mL of F-12K medium supplemented with 10% fetal bovine serum (FBS; ByProducts, Guadalajara, JAL, and MEX). The cells were incubated at 37°C in an atmosphere containing 95% O_2_ and 5% CO_2_ for 36 days. Upon reaching 90% confluence, the cells were reseeded into new dishes and exposed to a metal mixture (as detailed below), and the medium was changed every 3 days (72 h). Samples were collected every 6 days for subsequent analysis.

### 2.2. Abatement of the Antioxidant Barrier

**Figure 1:**
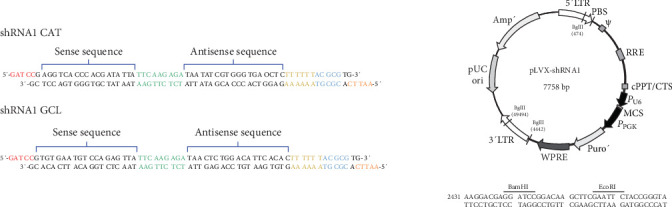
Sequences of the shRNAs designed based on the messenger RNA of the target sequence: ShRNA1 (AGGTCACCCACGATATTA) hybridizes from nucleotide 389–406 of catalase mRNA, shRNA2 (TGTGAATGTCCAGAGTTA) hybridizes to nucleotide 1900–1917 of GCLC mRNA and structure of plasmid pLVx-shRNA. GCLC, glutamate cysteine ligase catalytic.

Clone 9 cells were transfected with shRNAs designed to inhibit Cat and glutamate cysteine ligase catalytic (GCLC) expression. The shRNA sequences were specifically designed based on the target mRNA: ShRNA1 (AGGTCACCCACGATATTA) targeted nucleotides 389–406 of *Cat* mRNA, whereas shRNA2 (TGTGAATGTCCAGAGTTA) targeted nucleotides 1900–1917 of *GCLC* mRNA (Figure 1). These sequences were synthesized using IDTTM (Coralville, IA, USA). shRNAs were ligated into the pLVx-shRNA plasmid (Figure 1). Subsequently, *E. coli* (GCL-5a) cells were transformed with ligated plasmids via heat shock. Transformed cells were selected on a solid Luria–Bertani medium containing ampicillin (100 µg/mL). Positive colonies were expanded, the plasmids were purified, and the presence of the shRNA insert was confirmed using MluI (NEB) restriction enzyme digestion. Once confirmed, the plasmids were utilized to transfect Clone 9 cells using a 3 : 1 mixture of FuGENE 6 Transfection Reagent and DNA (Promega, Madison, WI, USA), followed by selection with puromycin (2 µg/mL). The cells were then harvested and expanded.

### 2.3. Exposure to the Mixture of As, Cd, and Pb

The following compounds were used for metal exposure: sodium arsenite (NaAsO_2_), cadmium chloride (CdCl_2_) (both from Aldrich Chemical Co. Inc., St. Louis, MO, USA), and lead acetate [Pb (C_2_H_3_O_2_)_2_·3H_2_O] (J.T. Baker, Mexico). Clone 9 cells were exposed to a metal mixture at final concentrations equivalent to occupationally relevant equimolar concentrations of NaAsO_2_ (2 µM), CdCl_2_ (2 µM), and [Pb (C_2_H_3_O_2_)_2_·3H_2_O] (5 µM). These concentrations are equimolar to those found in populations exposed to these metals. The treatment was refreshed with each medium change to maintain consistent metal mixture concentrations throughout the 36-day exposure period.

### 2.4. Cell Viability Measurement

Cell viability was assessed using the fluorescein diacetate [3′,6′-diacetylfluorescein] (FDA) method (Sigma–Aldrich, Inc., St. Louis, MO, USA), which is based on the activity of intracellular esterases that convert FDA into fluorescein, allowing for visualization via fluorescence microscopy. Cells were incubated with a solution containing 0.02 μg/mL ethidium bromide (EtBr) and 0.015 μg/mL FDA. Cell viability was evaluated using a fluorescence microscope (Olympus BMX-60 with a UM61002 filter); live cells appeared green, whereas dead cells stained red. A total of 100 cells were analyzed for each treatment, and the results are expressed as percentages.

### 2.5. Total RNA Extraction

Total RNA was extracted using the Maxwell simplyRNA Cell Kit (Promega). Cells were harvested using 0.25% trypsin, with 1 × 10^6^ cells collected for the assay. The cells were centrifuged at 1200 rpm for 2 min, and the supernatant was discarded. Approximately 200 μL of the homogenizing solution was added and vortexed. Subsequently, 200 μL of lysis solution was added and the mixture was placed in the cartridges provided by the kit. The automated purification protocol was performed using Maxwell equipment. RNA quantity was quantified using a NanoDrop ND-1000 spectrophotometer (Thermo Scientific). RNA quality was assessed by measuring the absorbance ratio at 260–280 nm, with the aim of achieving a ratio of 2.0 ± 0.15. Samples with lower purity were discarded, while suitable samples for polymerase chain reaction (PCR) were stored at −20°C.

### 2.6. Reverse Transcription (RT)–PCR

RT-PCR was performed using the access RT-PCR System kit (Promega) with 200 ng total RNA. The reaction mixture contained 5 μL AMV/Tfl 5X reaction buffer, 1 μL MgCl_2_, 1 μL dNTPs, 1 μL each sense, and antisense oligonucleotide (stock concentration at 10 mM; Table 1), 0.5 μL reverse transcriptase enzyme (RT-Pol), and 0.5 μL DNA polymerase enzyme. The final volume was adjusted to 25 μL using RNase-free water.

### 2.7. Immunofluorescence Staining

The cells were fixed with 4% paraformaldehyde for 20 min and washed with 1X PBS. Following fixation, cells were incubated with a blocking solution consisting of 5% BSA in PBS-Tween 20 for 1 h at room temperature. Cells were then incubated overnight at 4°C with primary antibodies against E-cadherin (CDH1; 1 : 250, GeneTex, Irvine, CA, USA), N-cadherin (CDH2; 1 : 250, GeneTex), *α*-smooth muscle actin (*α*-SMA; 1 : 500, GeneTex), VIM (1 : 100, Santa Cruz, Dallas, TX, USA), snail family transcriptional repressor 1 (Snai1; 1 : 250, GeneTex), and zinc finger e-box binding homeobox 1 (Zeb1; 1 : 250, GeneTex). After washing with 1X PBS, the cells were incubated with FITC-conjugated goat anti-mouse IgG or FITC-conjugated goat antirabbit IgG (1 : 500, Invitrogen, Waltham, MA, USA) for 2 h at room temperature. Nuclei were stained with 4,6-diamidino-2-phenylindole, dihydrochloride (1 : 500, Sigma, Darmstadt, Germany) for 10 min in the dark. Images were captured using a fluorescence microscope at 10x and 40x magnification.

### 2.8. Fluorescence Intensity Quantification

The fluorescence intensity was quantified using ImageJ software (NIH, USA). Each image was separated into individual channels, with the green channel considered the positive stain and the red channel as background. Images were divided into four quadrants, and the results were presented as the difference between the positive stain and background, normalized to the quantification area.

### 2.9. Wound Test

Cells were cultured to 85%–90% confluence and incubated for 24 h in F-12K medium with restricted FBS (2%) before wounding. Subsequently, a wound was created in the center of the dish using a 100 μL micropipette tip. The cells were washed with PBS to remove debris, and F-12K medium with serum restriction (2% SFB) was added. Wound closure was documented by capturing photographs at various time points (0, 4, 8, 12, and 24 h).

### 2.10. Statistical Analysis

Data are expressed as the mean ± standard error (SE). For immunofluorescence quantification, all measurements were repeated four times and are presented as mean ± standard deviation (SD). The Shapiro–Wilk test was used to assess data normality. For normally distributed data, statistical differences were evaluated using Student's *t*-test for comparisons between two groups or one-way analysis of variance (ANOVA) for multiple group comparisons, followed by Tukey's post hoc test. Statistical significance was defined as a one-tailed *p* − value  < 0.05. Data processing and analysis were performed using GraphPad Prism 7.0 (GraphPad Software, LLC).

## 3. Results

### 3.1. Abatement of the Antioxidant Barrier

**Figure 2:**
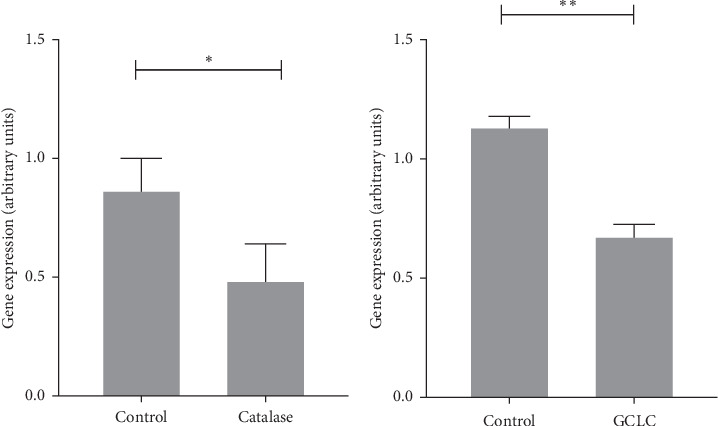
Transfection effectiveness. Downregulation of catalase and GCLC expression in C9 hepatic rat cells was measured 72 h after hairpin transfection. Student *t* test. *⁣*^*∗*^*p* < 0.05, *⁣*^*∗∗*^*p* < 0.01. GCLC, glutamate cysteine ligase catalytic.

To confirm that our model effectively induced a decrease in the antioxidant barrier, we measured the mRNA expression levels of both *Cat* and *GCLC* using RT-PCR. The results showed a significant decrease in *Cat* mRNA expression, with a reduction of 52% compared to that in the control group. For *GCLC*, a more pronounced decrease of 75% was observed, which was significantly different from that of the control (Figure 2).

### 3.2. Measurement of Viability

**Figure 3:**
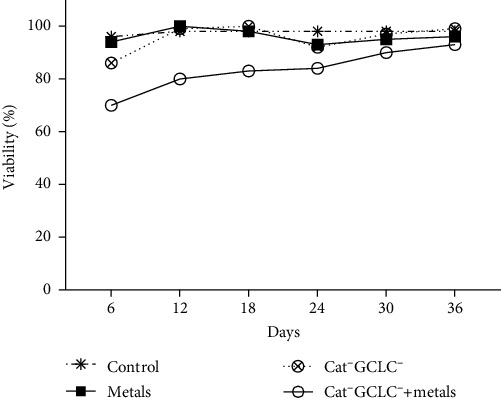
Viability measurement. For the measurement of viability, the dual fluorescein diacetate [3′,6′-diacetylfluorescein] (FDA) and ethidium bromide (EtBr) method was used. Viability was measured every 6 days until 36 days.

Cell viability was assessed using the FDA method. In cells treated with the metal mixture and Cat^−^GCLC^−^, the viability remained at ~20%, which was lower than that in the control. However, after 36 days, these cells showed signs of recovery of viability (Figure 3).

### 3.3. Expression of EMT Genes

**Figure 4:**
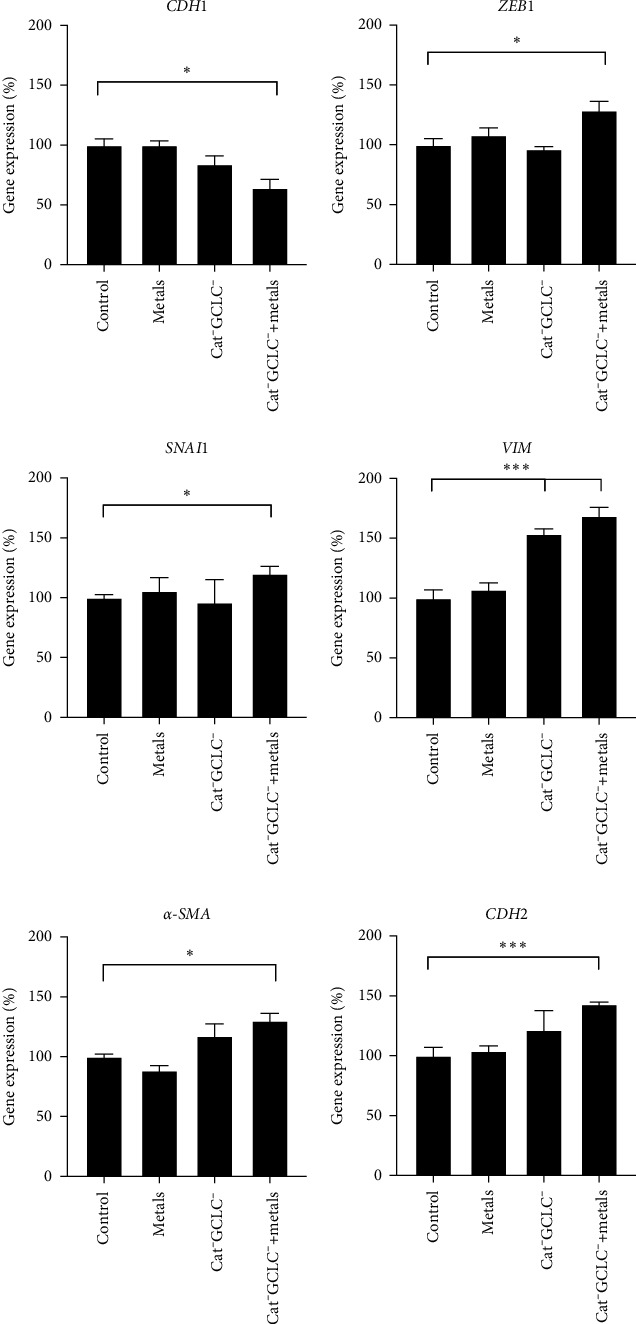
Changes in EMT-related genes. Changes expression in *CDH1*, *ZEB1*, *SNAI1*, *VIM*, *α-SMA*, and *CDH* were evaluated in C9 cells with a decreased antioxidant barrier. One-way ANOVA. *⁣*^*∗*^*p* < 0.05, *⁣*^*∗∗∗*^*p* < 0.001. ANOVA, analysis of variance; EMT, epithelial–mesenchymal transition; VIM, vimentin.

To investigate whether the reduction in the antioxidant barrier and/or treatment with the metal mixture induced a mesenchymal phenotype in the clone 9 epithelial cell line, we evaluated the expression of several genes. *CDH1* was used as an epithelial marker, whereas *ZEB1* and *SNAI1* served as transcription factors (TFs) involved in EMT. VIM, *α*-SMA, and CDH2 were used as the mesenchymal markers. Additionally, *CDH1* was downregulated in Cat^−^GCLC^−^+metal cells compared to that in the control group. We also observed a significant increase in the expression of both *ZEB1* and *SNAI1* in Cat^−^GCLC^−^+metal cells relative to the control. The most substantial change was noted in *VIM* expression, with evident alterations in both Cat^−^GCLC^−^ and Cat^−^GCLC^−^+metal cells. Mesenchymal markers, including *α-SMA* and *CDH2*, were overexpressed in Cat^−^GCLC^−^+metal cells (Figure 4).

### 3.4. Immunofluorescence Quantification EMT Genes

**Figure 5:**
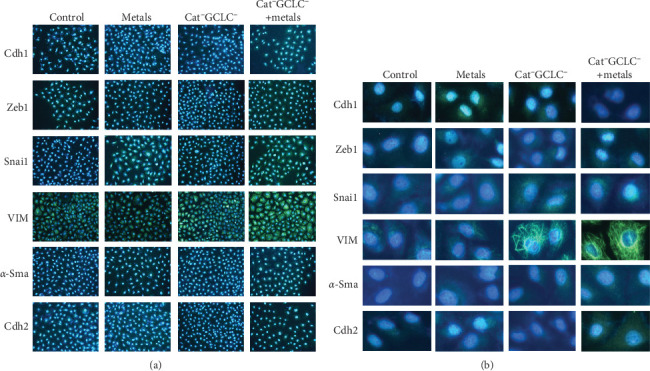
Immunofluorescence staining. A representative image of the cell population under immunofluorescence for each protein is shown in (A) (10X) and a more detailed image in (B) (40X). Images were taken under a Carl Zeiss fluorescence microscope.

Figure 5A presents a representative image of the cell population after immunofluorescence for each protein (10X), with a more detailed view shown in Figure 5B (40X). Quantitative analysis revealed an increase in CDH1 content in cells exposed to the metal mixture, likely attributable to As, which has been reported to enhance CDH1 expression at doses comparable to those used in this study [16]. Although depletion of the antioxidant barrier alone did not affect CDH1 expression, the combined effects of metal exposure and antioxidant barrier depletion led to a significant reduction in CDH1 expression.

**Figure 6:**
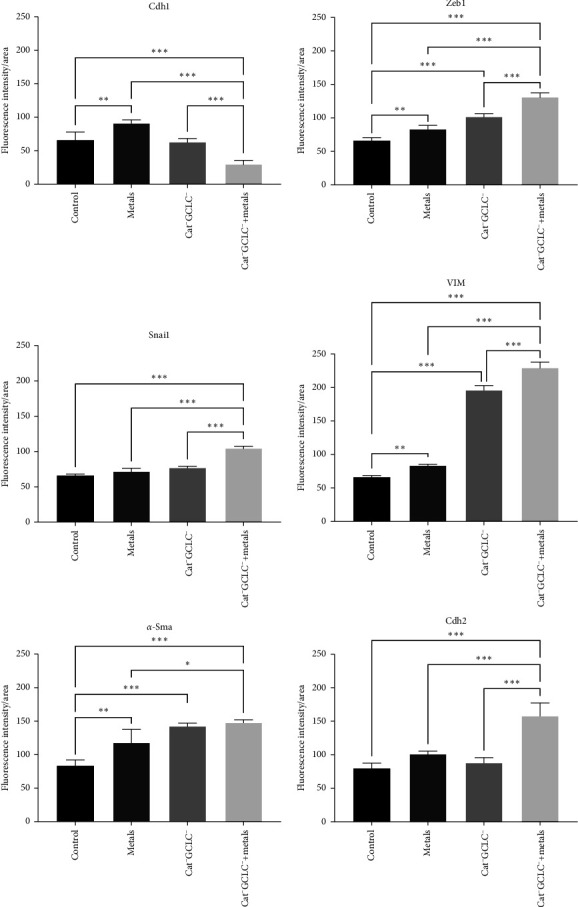
Fluorescence intensity quantification. The fluorescence intensity was determined using software ImageJ (NIH, USA). First, each image was split by channels, considering green channel as positive stain and red channel as background. The images were divided into four panels and the results were presented as the subtraction between positive stain and background divided by quantification area. Statistical differences among multiple groups were determined by one-way ANOVA and Tukey test as post hoc analysis. *⁣*^*∗*^*p* < 0.05, *⁣*^*∗∗*^*p*  < 0.01, *⁣*^*∗∗∗*^*p*  < 0.001. ANOVA, analysis of variance.

During EMT, epithelial cells typically lose their polygonal morphology and cell–cell adhesion, adopting fibroblast-like characteristics, including an elongated shape and increased expression of mesenchymal markers such as Vim and *α-Sma*. We evaluated the VIM content via immunofluorescence and observed a significant increase in cells lacking the antioxidant barrier, with even higher levels in cells exposed to the metal mixture (Figure 6).

Furthermore, *α-Sma* protein content showed a considerable increase relative to that in control cells, with the highest levels observed in cells lacking the antioxidant barrier, both alone and following treatment with the metal mixture. Furthermore, CDH2 content was twofold higher in cells without the antioxidant barrier and exposed to the metal mixture than in other treatments (Figure 6).

Given that *SNAI1* and *ZEB1* are regulators of the EMT, we assessed their protein levels. *SNAI1* levels increased in cells lacking the antioxidant barrier, displaying primarily perinuclear localization. In cells exposed to the metal mixture, *SNAI1* levels further increased and exhibited nuclear localization. Additionally, the expression levels of ZEB1 were elevated in all treatments relative to the control cells, with its nuclear localization being most prominent under metal mixture exposure (Figure 6).

### 3.5. Wound Test Evaluation

**Figure 7:**
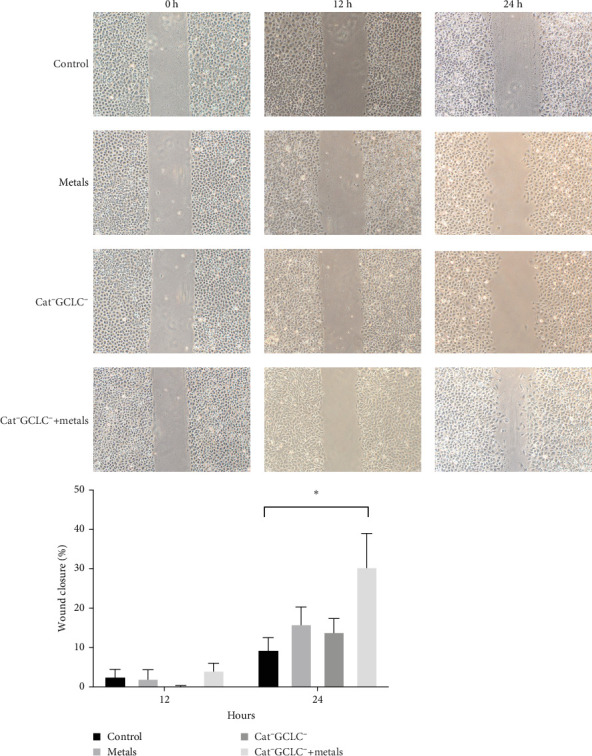
Evaluation of wound test. The evaluation of % wound closure was performed by quantifying the area with ImageJ software (NIH, USA). The percentage was measured at 12 and 24 h. Student's *t* test. *⁣*^*∗*^*p* < 0.05.

We conducted a wound healing assay (Figure 7) and observed that in Cat^−^GCLC^−^+metal-treated cells, migration commenced after 24 h, characterized by the presence of cells displaying elongated morphology. After 48 h, the wound was nearly closed, in sharp contrast to the control cells, where the wound area remained clearly visible.

## 4. Discussion

EMT is a well-characterized process in which epithelial cells lose their distinct characteristics and transform into mesenchymal cells, accompanied by the altered expression of cell adhesion molecules and changes in the cytoskeleton [17]. Although EMT is essential in nonpathogenic processes such as embryogenesis, it can be hijacked by epithelial cells during cancer development and progression [18].

Numerous studies have focused on the association between EMT and the initiation of tumor metastasis, as this program can provide epithelial cancer cells with traits necessary for migration to metastatic sites and the establishment of secondary tumors [19]. Thus, the ability to study and define the extent of EMT activation in individual cells is a valuable indicator of cancer aggressiveness and metastatic potential. EMT is dynamically regulated by a network of TFs, including SNAI1 and ZEB1, along with proteins associated with epithelial (CDH1) or mesenchymal (CDH2, VIM, *A*-SMA) properties [20].

Among the changes that occur during EMT, alterations in cell morphology and cell-matrix adhesion are crucial for transition. Loss of the epithelial marker CDH1 and acquisition of the mesenchymal marker VIM are considered pivotal events in this process [21].

We previously identified that decreased glutathione levels in the absence of oxidative stress can transiently inhibit actin-binding proteins, indicating that this stimulus is sufficient to induce changes in cell morphology via the actin cytoskeleton [22]. This suggests that a diminished antioxidant barrier promotes a state conducive to necessary changes in EMT; however, decreased barrier levels alone are not sufficient to establish EMT. In this study, we observed a significant decrease in CDH1 and a marked increase in VIM content in cells lacking an antioxidant barrier and exposed to a metal mixture, indicating a heightened metastatic potential.

Downregulation of *Cdh1* results in the breakdown of adherens junctions between cells, leading to a loss of cell polarity and acquisition of a mesenchymal phenotype with migratory capabilities [19]. Additionally, intermediate filaments, a major component of the cytoskeleton in mesenchymal cells, primarily consist of VIM. Increasing evidence suggests that aberrant expression of VIM in epithelial cancer cells facilitates the acquisition of invasive and metastatic properties; thus, VIM is considered a marker of EMT and is associated with poor chemotherapy response [16, 23].

Cytoskeletal structures and cell signaling pathways are disrupted in cells undergoing EMT. The activation of TFs such as SNAI1, SLUG, ZEB1, and TWIST is a key event that promotes EMT. Depending on the extent of EMT, these TFs can be expressed at levels sufficient to repress genes that regulate critical epithelial features or to induce the expression of mesenchymal markers [19].

SNAI1 levels increased upon exposure to the metal mixture; however, this localization was primarily perinuclear in nature. This phenomenon may be attributed to alterations in the WNT signaling pathway due to exposure to metals, known to inhibit this pathway [24], which may explain the perinuclear localization of SNA1, given that the WNT pathway regulates its localization [25]. However, further studies are required to explore this relationship. Furthermore, treatment of cells with a metal mixture lacking an antioxidant barrier resulted in increased nuclear expression of SNAI1. SNAI1 is a classic TF that modulates EMT in various tumor types. Numerous studies have clarified the molecular mechanisms by which SNAI1 regulates EMT, including the upregulation of matrix metalloproteinase genes such as MMP9, which degrade the basement membrane and stimulate tumor cell invasion [26]. Some studies have demonstrated that targeted downregulation of SNAI1 can reverse EMT [27], underscoring the correlation between increased SNAI1 levels and EMT promotion.

SNAI1 is a highly unstable protein characterized by rapid turnover. Various mechanisms exist that either enhance or inhibit nuclear import and export of SNAI1, with phosphorylation and post-translational regulation playing significant roles. Furthermore, a previous study demonstrated an association between WNT signaling and other SNAI1-mediated pathways in triggering EMT [28]. Nevertheless, any aberrations in nuclear transport mechanisms can disrupt the finely tuned balance of compartmentalization, thereby affecting SNAI1-mediated EMT signaling.

Conversely, ZEB1 suppresses the expression of components of the cell polarity complex and downregulates tight junction genes, promoting EMT while enhancing the expression of mesenchymal proteins CDH1 and VIM [16]. Recent data indicate that ZEB1 is a critical player in cancer progression, and its expression has been associated with poor differentiation, aggressive disease, metastatic development, and poor clinical prognosis across various cancer types [29]. Consistent with our results, we observed a clear increase in both ZEB1 gene and protein expression in cells lacking an antioxidant barrier and exposed to a metal mixture, which correlated with increased CDH2 and VIM protein levels.

In conclusion, the findings of this study indicate that the combination of a decreased antioxidant barrier and exposure to a mixture of metals, including As, Cd, and Pb, creates an environment that allows cells to acquire a mesenchymal-like phenotype characterized by greater plasticity and migratory capacity, as corroborated by the wound assay. Based on these findings, we propose that a decreased antioxidant barrier (glutathione and Cat) may serve as a risk biomarker in humans exposed to these metals. However, further studies in exposed populations are required to determine the utility of these potential biomarkers.

## Figures and Tables

**Table 1:** Detail of primers sequence of EMT involved genes.

Gene	Forward	Reverse
Cdh1 rat	AGACCAACGAGGGCATTCTG	TCAGCCCGAGTGGAAATGAC
Snai1 rat	TCCGATGAGGACAGTGGCA	CAGTGGGAGCAGGAGAAAGG
Zeb rat	AGCCAAATGGAAACCAGGATGA	AGGTCACATGCGTACATCCC
Cdh2 rat	GACCCAGAAGATGATGTAAG	CTCAGCGTGGATAGGC
*α*-SMA rat	TGCTAACAACGTCCTCTCGG	GAAAAGAACTGAAGGCGCTGA
VIM rat	TCCTTCGAAGCCATGTCCAC	GTGGTCACATAGCTCCGGTT

Abbreviation: EMT, epithelial–mesenchymal transition.

## Data Availability

All data are expressed in the manuscript, but if necessary, particular data will be available upon request from the corresponding author.
